# Degradation of Diazo Congo Red Dye by Using Synthesized Poly-Ferric-Silicate-Sulphate through Co-Polymerization Process

**DOI:** 10.3390/polym15010237

**Published:** 2023-01-03

**Authors:** Nor Fauziah Zainudin, Sung Ting Sam, Yee Shian Wong, Hanafi Ismail, Saparu Walli, Kazuki Inoue, Go Kawamura, Wai Kian Tan

**Affiliations:** 1 Faculty of Chemical Engineering & Technology, Universiti Malaysia Perlis (UniMAP), Arau 02600, Perlis, Malaysia; 2 Centre of Excellence Geopolymer and Green Technology (CEGeoGTech), Universiti Malaysia Perlis, Kompleks Pusat Pengajian Jejawi 2, Taman Muhibbah, Arau 02600, Perlis, Malaysia; 3 Faculty of Civil Engineering & Technology, Universiti Malaysia Perlis (UniMAP), Arau 02600, Perlis, Malaysia; 4 School of Materials and Mineral Resources Engineering, Engineering Campus, Universiti Sains Malaysia, Nibong Tebal 14300, Penang, Malaysia; 5 Frutania Industry Sdn. Bhd., Lot 1 & 2, Kawasan Industri MARA Jejawi, Jalan Kangar-Arau, Kangar 01000, Perlis, Malaysia; 6 Department of Electrical and Electronic Information Engineering, Toyohashi University of Technology, 1-1 Hibarigaoka, Tempaku-cho, Toyohashi 441-8580, Japan; 7 Institute of Liberal Arts and Sciences, Toyohashi University of Technology, Toyohashi 441-8580, Japan

**Keywords:** diazo Congo red, polymeric coagulant, co-polymerization, coagulation, wastewater

## Abstract

The ability of poly-ferric-silicate-sulphate (PFSS) synthesized via a co-polymerization process has been applied for the removal of diazo Congo red dye. A novel degradation pathway of diazo Congo red dye by using PFSS is proposed based on LC–MS analysis. Diazo Congo red dye was successfully removed using synthesized PFSS at lower coagulant dosages and a wider pH range, i.e., 9 mg/L from pH 5 to 7, 11 mg/L at pH 9, and 50 mg/L at pH 11. The azo bond cleavage was verified by the UV–Vis spectra of diazo Congo red-loaded PFSS and FTIR spectra which showed disappearance of the peak at 1584 cm^−1^ for –N=N– stretching vibrations. The synchronized results of UV–Vis spectra, FTIR, and the LC–MS analysis in this study confirmed the significance of the Si and Fe bond in PFSS towards the degradation of diazo Congo red dye. The successfully synthesized PFSS coagulant was characterized by FTIR, SEM, TEM, and HRTEM analysis. From this analysis, it was proven that PFSS is a polycrystalline material which is favorable for the coagulation–flocculation process. Based on all these findings, it was established that synthesized PFSS can be employed as a highly efficient polymeric coagulant for the removal of dye from wastewater.

## 1. Introduction

Different types of dyes have been extensively used in numerous industries, including the textile, printing, leather, paper, pulp, and cosmetic industries. The annual production of dyes has been estimated to be 70,000 tons and about 10–20% is lost during the dyeing process and finishing operations [1,2]. If these dye effluents are discharged directly into receiving water bodies without proper treatment, they can cause huge damage to the ecological system, reducing light penetration and, consequently, resulting in a serious threat to aquatic life and public health [3].

Azo dyes represent the largest group of synthetic dyes which account for 70% of the annual consumption of these compounds [4]. Azo dye pollutants are a major concern due to their color visibility and toxicity. Azo dyes are characterized by the azo bond (–N=N–) and their associated chromophores and auxochromes. These dyes are carcinogenic, mutagenic, and toxic to aquatic life and human beings [5]. Therefore, considerable attention has been focused on removing the residue of these dyes before they are discharged into the environment.

A number of treatment methods have been reported for removing azo dyes, for instance, biological [6], nanofiltration [7], photocatalytic [8,9], catalytic ozonation [10], adsorption [11,12,13], coagulation–flocculation [14], and Fenton [15] methods. Among these methods, the coagulation–flocculation method is well-established due to its low capital cost, ease of operation, and effective performance that is adaptable for high-concentration wastewater treatment [16,17].

Conventional metal salt coagulants such as aluminum sulphate (alum), ferric chloride (FeCl_3_), and ferric sulphate (Fe_2_(SO_4_)_3_) are widely used in the coagulation–flocculation process [18]. However, these coagulants suffer from limitations such as rapid formation of hydrolysis species during dilution that cannot be controlled [19]. Therefore, relatively new types of coagulants based on polymeric inorganic coagulants such as poly-ferric sulphate (PFS), poly-ferric chloride (PFC), poly-aluminum chloride (PAC), poly-aluminum ferric silicate chloride (PSiFAC), and poly-aluminum ferric sulphate (PAFS) have been developed [20,21]. However, Al-based coagulants are now doubtful due to the fact that lifetime cumulative aluminum intake can reportedly lead to the potential of Alzheimer’s disease [19].

In this study, poly-ferric-silicate-sulphate (PFSS) was synthesized using a co-polymerization method and rice husk ash as raw material. The synthesized PFSS coagulant was characterized by Fourier transform infrared (FTIR), scanning electron microscope (SEM), transmission electron microscope (TEM), and high-resolution transmission electron microscope (HRTEM) analyses. The coagulation process on diazo Congo red dye at various pH values and PFSS dosages was investigated. UV–Vis absorption (UVA) scanning, FTIR analysis, and liquid chromatography–mass spectrometry (LC–MS) analysis were performed to verify diazo Congo red degradation. Although polymeric inorganic coagulants are known to be able to treat dye solutions [22,23,24,25], to date, there is no report about the degradation mechanism pathway of diazo Congo red by PFSS, especially by self-made sodium silicate. Hence, the plausible degradation pathway of diazo Congo red dye was studied based on experimental findings.

## 2. Materials and Methods

### 2.1. Materials

Rice husk (RH) was collected from the BERNAS rice mill, Perlis, Malaysia. Sodium hydroxide (98%) and iron (II) sulfate heptahydrate (99.6%) were purchased from HmBG Chemicals (Hamburg, Germany). Sulfuric acid (98%) was obtained from Merck (Darmstadt, Germany). Sodium chlorate (≥99%) was supplied by Sigma-Aldrich (St. Louis, MO, USA), while sodium bicarbonate (99.7%) was obtained from Bendosen Laboratory Chemicals (Bendosen, Norway). Diazo Congo red dye was purchased from Acros Organic (Mumbai, India).

### 2.2. Synthesis of Sodium Silicate

In the present work, sodium silicate was synthesized by extracting silicate from rice husk ash. All the rice husk used in this research was ground and passed through a 600 µm sieve. Then, the rice husk was washed using distilled water to remove dust or impurities. After that, the RH was dried in an oven at 105 °C for 24 h [26]. The dried rice husk was then calcined at a heating rate of 5 °C/min up to 550 °C for 2 h.

Synthesis of sodium silicate was carried out using a modified methodology by Roschat et al. [27]. First, 50 g of rice husk ash (RHA) was dispersed into 500 mL of 2 M sodium hydroxide aqueous solution. Then, the mixture was heated and stirred at 350 rpm in a covered 500 mL Erlenmeyer flask at 90 °C for 1 h. Afterwards, the solution was cooled to room temperature after the boiling process. Then, the transparent solution of sodium silicate obtained was filtered using filter paper, while the remaining carbon residue on top of the filter paper was washed and dried at 105 °C for 24 h.

### 2.3. Synthesis of Metal Salt Solution

Metal salt solution was synthesized using a slightly modified method by Chen et al. [28]. First, 55.6 g of ferrous sulfate heptahydrate (FeSO_4_·7H_2_O) was dissolved in 45 mL of 20% sulfuric acid (H_2_SO_4_) solution under slow stirring in a plastic beaker at 60 °C to obtain a homogeneous ferrous sulfate liquid mixture. Then, 3.55 g of sodium chlorate (NaClO_3_) was added as the oxidant to oxidize the Fe(II) into Fe(III).

### 2.4. Synthesis of Poly-Ferric-Silicate-Sulphate Coagulant

In order to synthesize the PFSS coagulant, the synthesized sodium silicate was diluted to 3.0 wt.% with deionized water. Then, a calculated amount of the diluted sodium silicate with a Si/Fe ratio of 2.41 was introduced slowly into 4 mL of sulfuric acid solution (20%) under magnetic stirring at room temperature to obtain a poly-silicic acid solution (PS). The pH was regulated to pH 3.63 ± 0.2 by using 0.5 M H_2_SO_4_ and 1 M NaOH. The PS solution was aged for 31 min for effective polymerization. Next, the metal salt solution and PS solution were mixed and stirred rapidly at 63 °C. After 10 min, a measured amount of NaHCO_3_ powder with an OH/Fe ratio of 0.34 was added into the Fe(III) solution with a stirring rate of 100 rpm at the same synthesis temperature. The mixture was stirred for 60 min, and then was stored at room temperature for at least 180 min for further polymerization. The optimum conditions for PFSS synthesis, including Si/Fe ratio, OH/Fe ratio, pH, PS aging time, and PFSS polymerization temperature as mentioned above, were suggested by Design Expert Version 7.0 software (Stat-Ease Inc., Minneapolis, MN, USA) (data not shown).

### 2.5. Coagulation Experiments

The coagulation–flocculation experiments were performed using a jar testing apparatus (JLT6, VELP Scientifica, Usmate, Italy) at room temperature. Each beaker was filled with 250 mL of diazo Congo red solution. The initial pH was adjusted to pH values of 3, 5, 7, 9, and 11 by using 0.1 M H_2_SO_4_ and 0.1 M NaOH solution. The initial pH was measured using a HANNA pH meter (HI8424, HANNA Instruments, Woonsocket, RI, USA). PFSS coagulant dosages were added to the solutions, which were rapidly mixed at 200 rpm for 2 min to obtain a homogeneous dispersion, followed by slow mixing at 40 rpm for a duration of 15 min and sedimentation for 30 min. Water samples were collected from the beaker 2 cm below the water surface for further analysis. The subsequent experiments were conducted by varying the PFSS dosages from 3 to 11 mg/L (at pH 5), from 7 to 11 (at pH 7 and 9), and from 11 to 250 mg/L (at pH 11). The procedures continued as previously described.

### 2.6. Analysis of Degradation Products and the Synthesized PFSS Coagulant

In order to observe the successful degradation of diazo Congo red dye using PFSS coagulant, UV–Vis spectra in the wavelength range of 200–800 nm was also performed using a UV–Vis spectrophotometer (UV-1800, Shimadzu, Kyoto, Japan). The experiments were conducted in duplicate and the standard deviation was maintained below 3%. Fourier transform infrared (FTIR) analysis was performed to study the surface of the PFSS coagulant, diazo Congo red dye, and sludge obtained after coagulation by PFSS. The FTIR spectra were obtained using a FTIR spectrometer (Spectrum 65, Perkin Elmer, Waltham, MA, USA) in the range of 500–4000 cm^−1^ by following the potassium bromide (KBr) method. Diazo Congo red degradation was analyzed using LC–MS (TSQ Quantum Access MAX, Thermo Scientific, Waltham, MA, USA) equipped with an Agilent Eclipse Plus C18 (4.6 mm × 100 mm, 3.5 μm) column and at a flow rate of 250 µL/min. The morphologies of the synthesized PFSS were observed using a scanning electron microscope (SEM) (SU1510, Hitachi, Tokyo, Japan) and a high-resolution transmission electron microscope (HRTEM) (JEOL, JEM-2100, Tokyo, Japan).

## 3. Results and Discussion

### 3.1. Effect of pH

**Figure 1:**
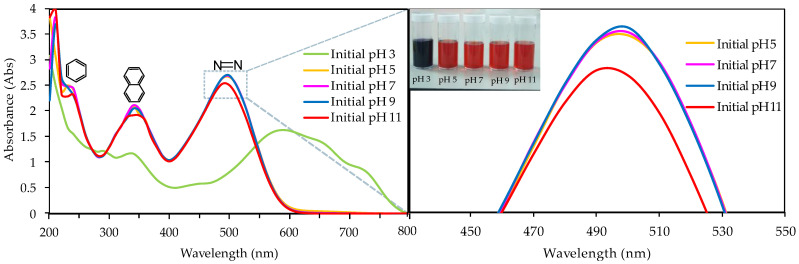
The UV–Vis spectra of diazo Congo red dye at different initial pH values. Inset: Photo of the dye solution at different initial pH conditions.

The spectra of diazo Congo red dye exhibited two major peaks at 342 nm and 498 nm which were attributed to the naphthalene rings (aromatic rings) and the π−π* transitions of the azo group (–N=N–), respectively. Meanwhile, the peak at 240 nm corresponded to the di-substituted benzene derivatives. The UV–Vis spectra of diazo Congo red dye at different initial pH values are demonstrated in Figure 1. The spectra were also measured to understand the effect of pH on the color of the dye solution. The UV spectra of the diazo Congo red dye show the absorbance value in a very acidic pH solution (pH 3); the color of diazo Congo red dye changes to blue–violet (see inset of Figure 1). This phenomenon might be due to the behavior of diazo Congo red which is a dipolar molecule –H_3_N^+^–R–SO_3_^–^ at a very acidic pH [29].

As clearly shown in Figure 1, the main band is different at an initial pH of 3 as compared with other initial pH values. The main band for diazo Congo red at an initial concentration of 50 mg/L and a pH range from 5 to 11 is 498 nm. However, at pH 3, the main band of the diazo Congo red solution gradually shifts to a longer wavelength, up to 590 nm. The intensity of the absorbance also decreases at an initial pH of 3. This might be attributed to the partial self-association of diazo Congo red dye which can be described as anionic dimers in face-to-face arrangement to minimize their hydrophobic interaction with water [30]. Consequently, the coagulation of diazo Congo red dye in this study was conducted only within the pH range from 5 to 11.

**Figure 2:**
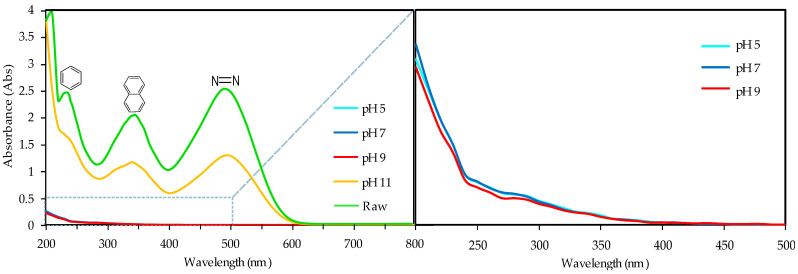
The UV–Vis spectra of diazo Congo red dye after the coagulation process at different initial pH values.

The pH of the solution is an important parameter that influences the removal of dye in wastewater. The influence of pH values ranging from 5 to 11 was studied and the results are reported in Figure 2. For these experiments, the initial concentrations of diazo Congo red and PFSS coagulant dosage were fixed at 50 mg/L and 11 mg/L, respectively. The decrease in the absorbance of the azo group at 498 nm and the naphthalene rings at 342 nm verified that the coagulation of diazo Congo red dye was successful [31]. Meanwhile, the increment in absorbance at 240 nm confirmed that the complex azo dye was changed into simpler phenyl components [32]. The coagulation process of the PFSS coagulant transformed the azo dyes into colorless aromatic amines due to absence of the –N=N– bond. These data were supported by the FTIR results which are further discussed in Section 3.3, and proved that the absence of a peak at 1584 cm^−1^ for the –N=N– stretching vibrations in the FTIR spectra of CR-loaded PFSS (after coagulation of diazo Congo red) was a result of cleavage of the azo bond.

This study reveals that acidic and basic environments have significant effects on the coagulation of diazo Congo red dye. As illustrated in Figure 2, the effective pH range for dye removal is wider, ranging from 5 to 9, and the maximum dye removal efficiency is completely achieved. In acidic conditions, the Fe ions in the solution begin to bond with hydroxyl ions to form polymeric species with a positive charge. These positive polymeric species can strongly combine with the anionic diazo Congo red dye and result in increased dye removal. According to Han et al. [16], diazo Congo red dye primarily exists in the form of azo in a solution and becomes stable at pH values greater than 5.

As the pH further increases above pH 9, the removal of dye decreases. In the basic condition, Fe ions mainly exist in the form of $\mathrm{Fe}{\left(\mathrm{OH}\right)}_{4}^{-}\;$ and contribute to re-stabilization of the dye solution. In addition, the negatively charged diazo Congo red dye and $\mathrm{Fe}{\left(\mathrm{OH}\right)}_{4}^{-}\;$ repel each other, decreasing the capability of coagulant and, consequently, reducing the dye removal efficiency [16].

### 3.2. Effect of Coagulant Dosage

**Figure 3:**
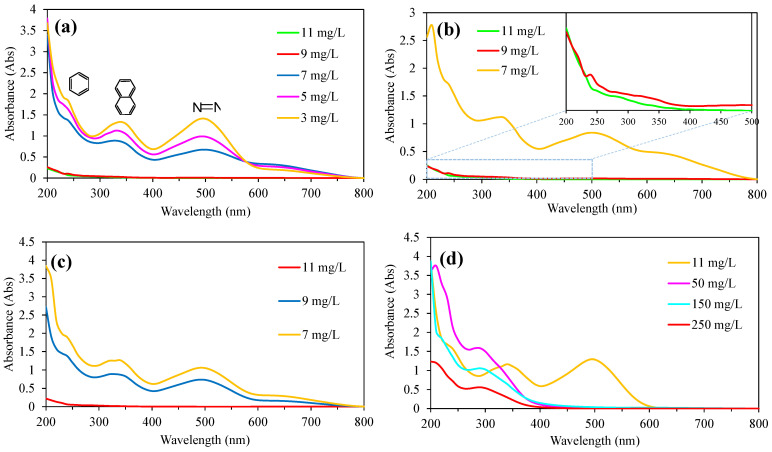
UV–Vis spectra analysis of diazo Congo red dye after treatment with different dosages of PFSS at: (**a**) pH 5; (**b**) pH 7; (**c**) pH 9; (**d**) pH 11.

The effect of the coagulant dosage on the coagulation of diazo Congo red dye at different pH values was studied in this work and the results are demonstrated in Figure 3. A number of studies have indicated that coagulation performance efficiency initially increased with the coagulant dosage, and then decreased at high values of coagulant dosage [33,34,35]. This can be rationalized in terms of higher charge neutralization capacity and bridge-aggregation ability. As can be clearly seen in Figure 3, although the coagulation performance increases with the coagulant dosage, it can be observed that above the critical value of 7 mg/L, coagulation efficiency becomes independent of the coagulant dosage. This is mainly due to the reversal of particles’ surface charge, which leads to re-stabilization of colloidal particles during overdosing. The same trend was observed in the research conducted by Wei et al. [19], where an optimum coagulant of 16 mg/L was achieved at pH 7.67.

From the UV–Vis spectra analysis of the treated diazo Congo red dye in Figure 3, it can be observed that the initial pH of the diazo Congo red solution has a significant impact on the coagulant dosage required to treat the dye. A small dosage was required to completely remove the diazo Congo red dye at a lower initial pH of the solution. However, at pH 11, at least about 50 mg/L of PFSS coagulant was needed to completely remove the diazo Congo red dye from the solution.

### 3.3. FTIR Analysis

**Figure 4:**
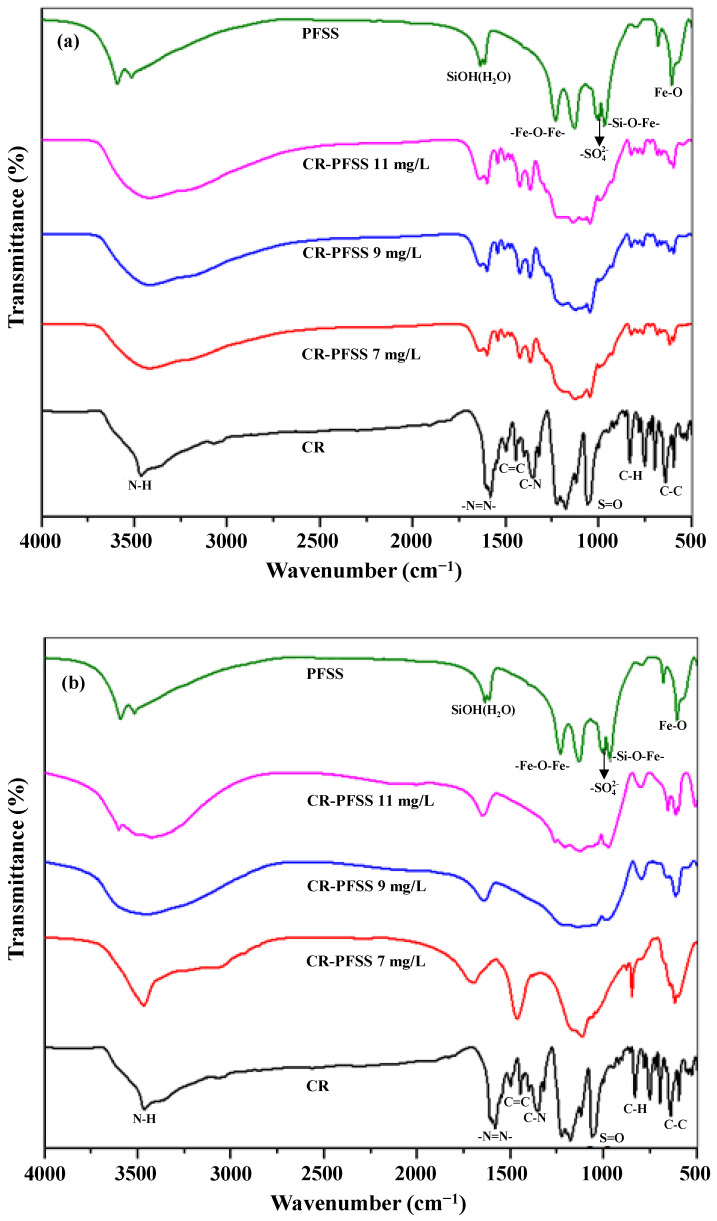
FTIR spectra of PFSS before and after degradation of diazo Congo red dye at: (**a**) pH 5; (**b**) pH 11.

Figure 4 demonstrates the FTIR spectra of PFSS, CR, and CR-loaded PFSS at different dosage loadings and pH values. The PFSS spectra show two bonds at 3200–3650 cm^−1^ and 1637 cm^−1^, which can be assigned to the stretching vibrations of –OH and to the bending vibrations of water absorbed, polymerized, or crystallized in the coagulants [19]. The presence of a peak at 1622 cm^−1^ can be attributed to SiOH hydration. Meanwhile, there are absorption peaks around 1130–1230 cm^−1^, which are related to the asymmetric stretching vibrations of Fe–O–Fe. Furthermore, there is an absorption peak at 997 cm^−1^, which can be attributed to the ${\mathrm{SO}}_{4}^{2-}$ stretching vibration. The absorption peak at 967 cm^−1^, associated with Si–O–Fe, proves the formation of a high polymeric substance of PFSS, which is favorable for the coagulation–flocculation process [36,37]. The peaks at 800 cm^−1^ and 600 cm^−1^ can be attributed to Si–O and stretching vibrations of Fe–O, respectively.

The spectra of diazo Congo red dye exhibited a peak for the N–H stretching vibrations of secondary amide at 3465 cm^−1^. The absorption peak at 1584 cm^−1^ represents –N=N– stretching vibrations and the peak at 1446 cm^−1^ can be attributed to aromatic C=C stretching vibrations. Meanwhile, the presence of peaks at 1355 cm^−1^ and 1063 cm^−1^ can be attributed to C–N bending vibrations and S=O stretching vibrations of sulfonic acid, respectively. In addition, the peaks observed at 832 cm^−1^, 698 cm^−1^, and 640 cm^−1^, can be attributed to C–H ring vibrations and stretching vibrations, as well as C–C bending vibrations which support the existence of aromatic rings. Meanwhile, the spectra of CR-loaded PFSS (after coagulation) show the disappearance of the peak at 1584 cm^−1^ for –N=N– stretching vibrations, which is evidence of azo bond cleavage. In addition, the shift of peaks within 1622–1640 cm^−1^ and reduction in their amplitude (at different PFSS-loaded dosages) reveal that diazo Congo red dye reacts with SiOH hydration. In addition, the strong N–H peak in diazo Congo red dye was diminished after coagulation using PFSS coagulant. However, in alkaline medium, this strong peak was obviously diminished after an increase in the dosage of PFSS up to 50 mg/L. In the meantime, increasing the PFSS dosage from 50 to 150 mg/L also resulted in the appearance of a broad peak at 1090 nm^−1^. This could be caused by the combined action of Fe–O–Fe and Si–O–Fe functional groups [38,39].

### 3.4. Morphological Analysis

**Figure 5:**
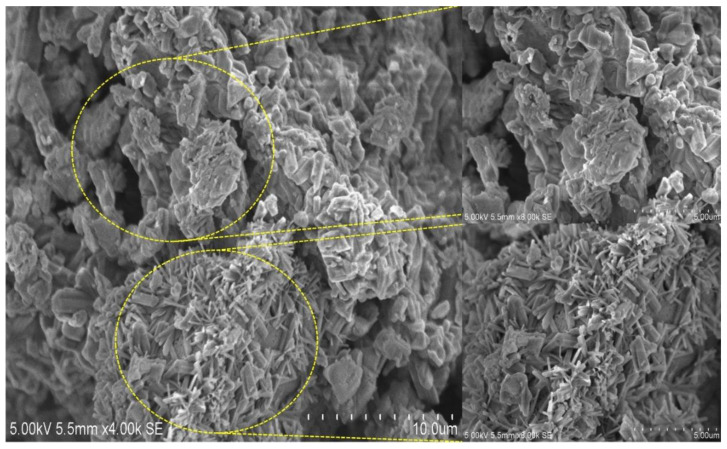
SEM image of the synthesized PFSS.

In this work, the morphological characteristics of PFSS were investigated by SEM, TEM, and HRTEM. In Figure 5, the appearance of two different morphologies of synthesized PFSS can be observed in the SEM images. Some of the synthesized PFSS consists of irregular granule units and there is aggregation among the granules that make up a porous structure, while the other synthesized PFSS consists of a multilayer of three-dimensional tube-like features, which shows the good crystallization process of PFSS. The porous structure of the synthesized PFSS was due to the hydrolysis of ferric ions. The irregularity of the synthesized PFSS coagulant corresponds to the fractal dimension, which relates to an increase in coagulation efficiency in this study [28,40].

**Figure 6:**
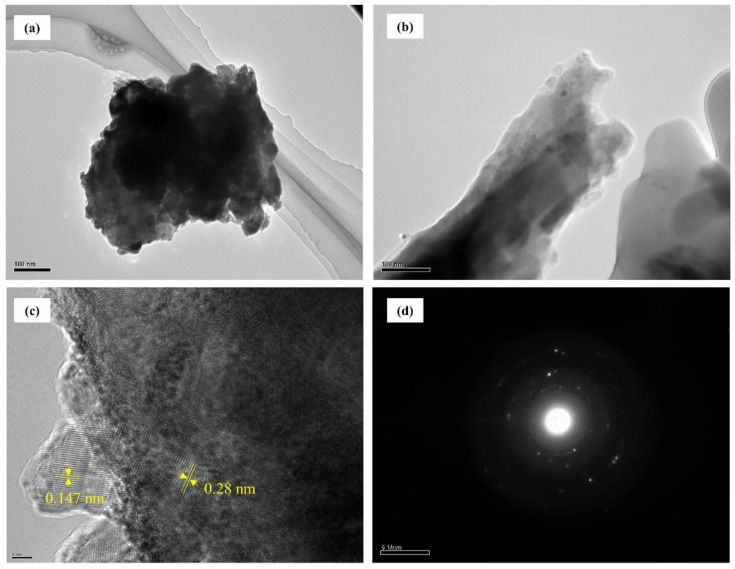
(**a**,**b**) TEM images; (**c**) HRTEM image; (**d**) SAED pattern of the synthesized PFSS.

For more in-depth insight into the structure of the synthesized PFSS, detailed investigations by TEM and HRTEM were conducted. As shown in Figure 6a, the PFSS demonstrates light agglomerate irregular particles. However, rod-like morphologies can also be observed in the PFSS image depicted in Figure 6b. This finding was in agreement with the SEM result. Figure 6c shows a HRTEM image of PFSS coagulant. A randomly oriented interplanar can be observed in the HRTEM image shown in Figure 6c, which reveals the existence of a polycrystalline structure in the PFSS coagulant [41,42]. The interplanar spacing of 0.147 nm and 28 nm belong to silicon dioxide and iron hydroxide, respectively. The polycrystalline structure of the synthesized PFSS is confirmed by the appearance of a bright spot along the ring in the selected-area electron diffraction (SAED) pattern, as depicted in Figure 3d.

### 3.5. Degradation Pathway of Diazo Congo Red Dye

Research was further conducted to investigate the degradation pathway of diazo Congo red dye. FTIR and UV–Vis spectrum analyses were performed to verify the degradation pathway. UV–Vis spectrum scanning from 200 to 800 nm was conducted to study the molecular structure changes in diazo Congo red dye. As previously discussed in Section 3.1, the visible region located at 498 nm for diazo Congo red dye was associated with the azo linkage. Additionally, the peaks in the UV region, which were at 342 nm and 240 nm, corresponded to the naphthalene and benzene rings of the dye (aromatic rings) and di-substituted benzene derivatives, respectively [32]. As the coagulation takes place, the visible band of the dye decreases gradually. Simultaneously, a small deterioration of the band at 342 nm was observed, due to the opening of the naphthalene ring.

As previously discussed, pH has a strong influence on the coagulation efficiency of diazo Congo red dye. In an acidic environment, ${\mathrm{Fe}}^{3+}$ is hydrolyzed and polymerized under the bridging of hydroxyl groups (–OH) [43,44]. This large positively charged polymeric coagulant stabilizes the negatively charged dye solution through electrostatic interactions and, hence, improves the coagulation process. The coagulation of diazo Congo red dye has also been achieved via ion exchange, where the hydroxyl groups in PFSS molecules could be replaced by the negatively charged sulfonate groups (${\mathrm{SO}}_{3}^{-}$) in diazo Congo red anionic dye [29]. The addition of poly-silicic acid in poly-ferric coagulant by the co-polymerization method enhanced the coagulation process of diazo Congo red dye due to its netting–bridging effect. In addition, the presence of poly-silicic acid in the polymeric coagulant increased the polymerization degree of the coagulant, which could exhibit superior coagulation efficiency by rapid precipitation. Moreover, the covalent bond formed between the nitrogen atom in diazo Congo red dye and the O=S=O improved the stability of the PFSS coagulant and enhanced the coagulation behavior of the coagulant [29,45]. In the meantime, sulphate ions aid the coagulation process by accelerating the precipitation by linking the hydroxylated Fe polymers [46].

**Scheme 1:**
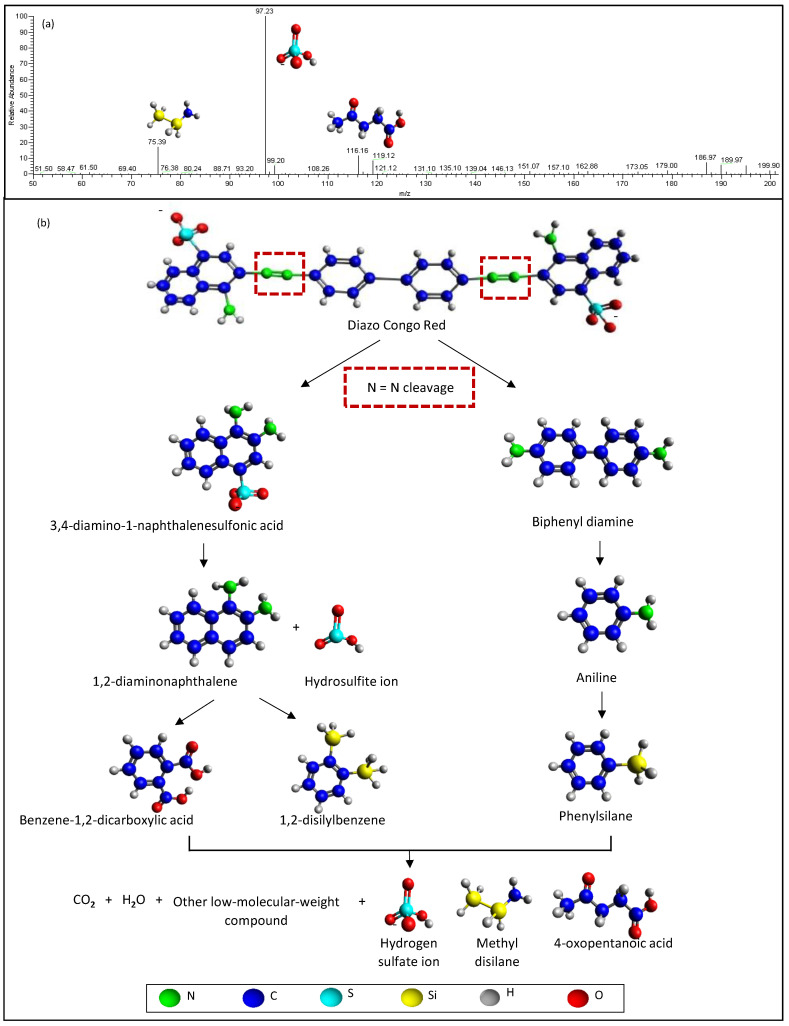
(**a**) LC–MS spectra; (**b**) The plausible degradation pathway of diazo Congo red by PFSS coagulant.

Scheme 1a shows the LC–MS analysis of degraded diazo Congo red solution. From the spectra, it can be observed that methyl disilane, 4-oxopentanoic acid, and hydrogen sulfate ions have lower mass fragmentation resulting from the degradation of complex diazo dye molecules.

Scheme 1b demonstrates the plausible degradation pathway of diazo Congo red solution by using PFSS coagulant. The degradation pathway of diazo Congo red dye was initiated by the cleavage of an azo bond which led to the formation of two molecules of 3,4-diamino-1-naphthalenesulfonic acid and biphenyl diamine. After that, 3,4-diamino-1-naphthalenesulfonic acid subsequently underwent a desulfonation reaction. Detachment of the sulfonate group from 3,4-diamino-1-naphthalenesulfonic acid tended to form 1,2-diaminonaphthalene and a hydrosulfite ion (–${\mathrm{SO}}_{3}H$), which was further decomposed to become sulfanide ions that were more stable [47]. Subsequently, 1,2-diaminonaphthalene underwent reactions which led to the formation of benzene-1,2-dicarboxylic acid (phthalic acid) and 1,2-disilylbenzene. In addition, biphenyl diamine was further decomposed to aniline. Then, aniline underwent successive deamination with silication reactions to form phenylsilane. All the intermediates that could, underwent further reactions and transformed into lower-molecular-weight compounds such as methyl disilane, 4-oxopentanoic acid, and hydrogen sulfate ions. These compounds could also be mineralized to carbon dioxide and water. This result is in agreement with the results shown by the analysis of FTIR and UV–Vis spectra. All the analyses conducted were found to provide strong evidence for the degradation of diazo dye by PFSS.

### 3.6. Comparative Studies of PFSS in the Present Study with Other Polymeric Inorganic Coagulants in Dye Wastewater Treatment

A review of the studies dealing with the application of different polymeric inorganic coagulants in reactive blue [25,48,49], reactive yellow [48], disperse yellow [25], Congo red [23], acid red [22], and simulated dyeing wastewater [50,51] removal published from 2011 to 2020 is presented in Table 1.

Most studies on polymeric inorganic coagulants have focused on parameters that affected the polymeric coagulant preparation methods as well as process parameters in dye coagulation and several have reported roughly on the mechanisms involved. However, none of these studies have reported on the degradation of dyes. The synchronized results of the UV–Vis spectrum analysis, FTIR analysis, and LC–MS analysis in the present study confirmed the success of the cleaved diazo bond and transformation of the dye molecules into the simplest structures such as methyl disilane, 4-oxopentanoic acid, and hydrogen sulfate ions. From these findings, for the first time, we proposed a plausible diazo Congo red degradation pathway using PFSS.

## 4. Conclusions

The synthesized PFSS successfully removed the diazo Congo red dye at a lower coagulant dosage in a wider pH range, which was 9 mg/L from pH 5 to 7, 11 mg/L at pH 9, and 50 mg/L at pH 11. The plausible degradation pathway for diazo Congo red dye coagulation was established. The UV–Vis spectra of diazo Congo red-loaded PFSS (after coagulation) and FTIR spectra showed the disappearance of the peak at 1584 cm^−1^ for –N=N– stretching vibrations, which confirmed the azo bond cleavage. The significance of the Si and Fe bond in PFSS towards diazo Congo red degradation was verified through the breakage of the azo bond and the formation of the simplest structures of products was proven by LC–MS analysis. In order to demonstrate the successful synthesis of PFSS, the structure and morphology characterization of the synthesized PFSS was investigated by conducting FTIR as well as SEM and TEM analyses. Based on the FTIR characterization results of PFSS, it can be concluded that a complex polymeric compound was formed in the synthesized PFSS coagulant. Good interactions between poly-silicic acid and the hydrolyzed ferric ions lead to the formation of Si–O–Fe bonds which are favorable in the coagulation process. The SEM images demonstrated that two different morphologies were found in the synthesized PFSS including an irregular and porous structure and three-dimensional tube-like features. The HRTEM analysis revealed the existence of a polycrystalline structure in the PFSS coagulant. As a result, the application of PFSS in dye removal from wastewater proved that it is an efficient polymeric coagulant. Furthermore, utilization of abundant rice husk ash could also overcome the disposal problem of this agricultural waste.

## Figures and Tables

**Table 1:** Comparison of the PFSS in this study with other polymeric inorganic coagulants in the coagulation of dye solution.

Polymeric Inorganic Coagulant	Dye Pollutant	Condition			Removal (%)	Degradation (Supported by Analysis)	Studies
		pH	Dosage (mg/L)	Initial Concentration (mg/L)			
Poly-ferric-silicate-sulphate (PFSS)	Diazo Congo red	5–9	11	50	100%	Degradation study (Proved by LC-MS analysis)	Present study
Poly-ferric chloride (PFC);	Reactive blue 19	9	350	150	92.37%	-	[49]
Poly-titanium tetrachloride (PTC)		7	350	200	99.83%	-	
Poly-aluminum chloride;	Acid red 119	3.42	4.55 g/L	140	94.1%	-	[22]
Poly-aluminum chloride sludge		3.8	57	140	95.25%	-	
Polymeric iron sulfate (PFS);	Congo red	6.35	141.66	77.82	98.28%	-	[23]
Ferrous sulfate;		10	159.64	50.24	96.14%	-	
Aluminum sulfate		8.26	80	187.92	84.37%	-	
Magnesium silicate polymer (MgSiPC)	Reactive Yellow 2;	12.08	62	100	96%	Mechanism study (No supported analysis)	[48]
	Reactive blue 2	12	78	100	100%		
Poly-magnesium silicate chloride (PMSC)	Simulated dyeing wastewater	12	40	-	82.8%	-	[50]
Poly-ferric(III) magnesium(II) sulfate (PFMS); poly-ferric sulfate (PFS); poly-ferric chloride (PFC); poly-aluminum sulfate (PAS)	Textile wastewater	8.3	280	-	PFMS = 88% PFS = 57% PFC = 62% PAC = 80%	-	[51]
Poly-aluminum chloride (PACl);	Reactive Cibacron Blue F3GA (RCB)	7.5	50	-	92%	-	[25]
	Disperse Terasil Yellow W-4G (DTY)	3	20	-	93%	-	
Poly-aluminum chloride poly(3-acrylamido-isopropanol chloride) (PACl-PAMIPCl)	RCB	7.5	50	-	95%	-	
	DTY	3	20	-	96%	-	

## Data Availability

The data presented in this study are available on request from the corresponding author.
